# Quantification and clinical relevance of gene amplification at chromosome 17q12-q21 in human epidermal growth factor receptor 2-amplified breast cancers

**DOI:** 10.1186/bcr2824

**Published:** 2011-02-02

**Authors:** Pierre-Jean Lamy, Frédéric Fina, Caroline Bascoul-Mollevi, Anne-Claire Laberenne, Pierre-Marie Martin, L'Houcine Ouafik, William Jacot

**Affiliations:** 1 Laboratoire de Biologie Spécialisée et d'Oncogénétique, Centre Régional de Lutte contre le Cancer Val d'Aurelle-Paul Lamarque, 208, rue des Apothicaires, Montpellier F-34298, France; 2 Laboratoire de Transfert d'Oncologie Biologie, Assistance Publique, Hôpitaux de Marseille, Boulevard Pierre Dramard, Marseille F-13916, France; 3 Unité de Biostatistique Centre Régional de Lutte contre le Cancer Val d'Aurelle-Paul Lamarque, 208, rue des Apothicaires, Montpellier F-34298, France; 4 UMR 911 CRO2: Centre de Recherche en Oncologie biologique et Oncopharmacologie, Boulevard Pierre Dramard, Marseille F-13344, France; 5 Service d'Oncologie Médicale, Centre Régional de Lutte contre le Cancer Val d'Aurelle-Paul Lamarque, 208, rue des Apothicaires, Montpellier F-34298, France

## Abstract

**Introduction:**

Human epidermal growth factor receptor 2 (HER2)-amplified breast cancers represent a tumor subtype with chromosome 17q rearrangements that lead to frequent gene amplifications. The aim of this study was to quantify the amplification of genes located on chromosome 17q and to analyze the relations between the pattern of gene amplifications and the patients' characteristics and survival.

**Methods:**

Patients with HER2-positive breast tumors (HER2 score of 3+ by immunohistochemistry or positive for *HER2 *amplification by fluorescence *in situ *hybridization (FISH)) (*n *= 86) and with HER2-negative breast tumors (*n *= 40) (negative controls) were included in this study. Using a quantitative polymerase chain reaction method and DNA extracted from frozen tumor specimens, 11 genes (*MED1*, *STARD3*, *HER2*, *GRB7, THRA, RARA*, *TOP2A*, *IGFBP4*, *CCR7*, *KRT20, KRT19 *and *GAS*), which are localized within Chr17q12-q21 and have a putative role in breast cancer development, were quantified. Relapse-free and overall survival rates were estimated from the date of surgery to the date of the event of interest (recurrence or death) using the Kaplan-Meier method.

**Results:**

Gene amplification was observed only in HER2-positive tumors, and the frequency of amplification decreased with the distance of the gene from *HER2*. *HER2 *presented the highest level of amplification. *TOP2A *was not included in the smallest region of amplification involving *HER2*. Amplification of *RARA*, *KRT20 *and *KRT19 *was significantly associated with node-positive breast cancer (*P *= 0.030, *P *= 0.002 and *P *= 0.033, respectively). During a median follow-up period of 55 months (range, 6 to 81 months), the subgroup of patients with hormone receptor-negative cancer and without *TOP2A *amplification showed the worst survival (relapse-free survival: hazard ratio (HR) = 0.29, 95% confidence interval (95% CI), 0.13 to 0.65, *P *= 0.001; and overall survival: HR = 0.28, 95% CI, 0.10 to 0.76, *P *= 0.008).

**Conclusions:**

*HER2 *amplification seems to drive genomic instability along chromosome 17q, leading to different patterns of gene amplification. This study confirms the clinical importance of identifying, among patients with HER2-positive breast tumors, the subgroup of patients with hormone receptor-negative and nonamplified *TOP2A *cancers as they have the worst prognosis.

## Introduction

Gene amplification at chromosome 17q (Chr17q) involves the human epidermal growth factor receptor 2 (*HER2*) gene and is observed in about 15% to 20% of breast cancers. *HER2 *amplification is associated with aggressive tumor behavior (increased metastatic potential and reduced survival), and it also predicts a patient's response to trastuzumab therapy [1,2]. Although HER2-positive breast cancers are classified as a tumor subgroup, they are quite heterogeneous. Indeed, they can be divided more or less equally into hormone receptor-negative (estrogen receptor/progesterone receptor-negative, or ER/PR-negative) and hormone receptor-positive (ER/PR-positive) tumors. In the ER/PR-positive subgroup, development of resistance to antihormonal therapy could be explained by the cross-talk between the HER2 and ER pathways [3]. Furthermore, first-line treatment of HER2-positive breast cancers with trastuzumab presents response rates that vary between 25% and 35%, and the benefit of trastuzumab therapy has been shown only in patients with high levels of *HER2 *amplification [4]. Comparative genomic hybridization arrays and fluorescence *in situ *hybridization (FISH) studies have shown that HER2-amplified breast cancers present a variety of alterations on Chr17 and gene amplifications at Chr17q12-q21 are particularly frequent and complex [5]. Among the genes that are located in this region and have been reported to be amplified in breast cancers, topoisomerase IIα (*TOP2A*), a gene coding for the enzyme targeted by anthracyclines, is located 2 Mb from *HER2*. *TOP2A *amplification might be linked to the sensitivity to anthracyclines observed in HER2-positive breast tumors [6-18]. Most of the published data indicate that *TOP2A *amplification occurs only in HER2-amplified tumors. However, some authors have reported cases in which *TOP2A *was amplified also in the absence of *HER2 *amplification [19,20]. Interestingly, the same studies do not support the role of *TOP2A *amplification as a predictive marker for response to anthracyclines. These works also report a different frequency of *TOP2A *amplifications and deletions. The presence of *TOP2A *deletions, often analyzed together with *TOP2A *amplification, has complicated the question of the relations among *TOP2A *alteration, prognosis and response to anthracyclines. Technical issues, the cutoff levels used to classify a gene as amplified or deleted and different quality assurance guidelines could also explain these discrepancies [21,22].

Nevertheless, the molecular variations within the Chr17q amplicon and their clinical implications remain largely unknown. Indeed, besides *TOP2A*, other genes which are located not only in the *HER2 *smallest region of amplification (SRA) [23,24] but also all along the long arm of the Chr17 [5,24-27] might also be amplified, and they could play functional roles in breast cancer development and progression. To explore this hypothesis, we carried out a quantitative analysis of a set of 11 genes that were chosen for their localization along Chr17q12-q21 and for their putative role in breast cancer development. Two genes were located on the centromeric side (*MED1 *and *STARD3*), and nine were located on the distal side (*GRB7*, *THRA*, *RARA*, *TOP2A*, *IGFBP4*, *CCR7*, *KRT20*, *KRT19 *and *GAS*) of Ch17q12-21 relative to *HER2 *localization. The relation between gene amplification and patients' characteristics and survival was then analyzed. We show that amplification of *TOP2A *and of other genes distal to *HER2 *does not occur in tumors in which *HER2 *is not amplified. We confirm that *TOP2A *is not included in the SRA involving *HER2 *and describe different copy number alterations along the distal side of Chr17q. We also show that *TOP2A *nonamplification is related to worse survival in the subpopulation patients with of ER/PR-negative tumors.

## Materials and methods

### Patients' characteristics, tumor samples and treatment

A total of 759 consecutive breast cancer patients referred to the Val d'Aurelle Cancer Center between March 2002 and May 2005 were prospectively entered into the database of a tumor DNA bank. Ethical approval was provided by the local research ethics committee. All patients gave their written, informed consent. The DNA bank was created using frozen, histologically proven invasive breast cancer specimens that were primarily handled for ER testing by the dextran charcoal method. Patients with primary HER2-positive breast cancer were initially selected on the basis of the level of HER2 expression by immunohistochemistry (IHC) using the HercepTest (DakoCytomation, Trappes, France). HER2 scores of 0 and 1 were considered negative. HER2 scores of 2+ were then checked by FISH for *HER2 *copy number (PathVysion HER-2 *DNA *Probe Kit; Abbott Molecular, Rungis, France). Only patients with HER2 scores of 3+ derived by IHC or positive for *HER2 *amplification by FISH were considered HER2-positive. *HER2 *copy number and Chr17 polysomy were then assessed by quantitative polymerase chain reaction (qPCR) as previously described [28]. Patients presenting with Chr17 polysomy without true *HER2 *amplification were excluded from the study. Adjuvant treatment was discussed in multidisciplinary medical meetings according to local and international guidelines and submitted for each patient's approval. All patients were treated at least by adjuvant radiation therapy and received anthracycline-based adjuvant therapy according to the medical standards at the time of diagnosis. Considering the patient accrual period, and despite their HER2-positive status, women were not treated with adjuvant anti-HER2 therapy, with the exception of patients included in a clinical trial to assess the benefits of trastuzumab as adjuvant therapy. The clinicopathologic characteristics and treatments are summarized in Table 1. Patients received regular follow-up examinations, that is, every 6 months for the first 5 years, then yearly after that.

**Table 1:** Patients' characteristics^a^

Characteristic	Number of patients (%) (*n* = 86)
Age, yr	
<50	33 (38.4%)
≥50	53 (61.6%)
T	
1	35 (40.7%)
2	46 (53.5%)
3	4 (4.6%)
4	1 (1.2%)
N	
N^+^	51 (59.3%)
N^-^	35 (40.7%)
SBR	
2	36 (42.3%)
3	49 (57.7%)
Missing	1
Hormone receptor status	
ER	
ER^-^	60 (69.8%)
ER^+^	26 (30.2%)
PR	
PR^-^	44 (51.2%)
PR^+^	42 (48.8%)
ER/PR	
ER^-^/PR^-^	42 (48.9%)
ER^-^/PR^+^	18 (20.9%)
ER^+^/PR^-^	2 (2.3%)
ER^+^/PR^+^	24 (27.9%)
Treatment	
Chemotherapy	
Anthracyclines	42 (48.8%)
Anthracyclines and taxanes	26 (30.2%)
No chemotherapy	18 (21.0%)
Radiation therapy	86 (100.0%)
Antihormone therapy	34 (39.5%)
Trastuzumab	20 (23.3%)
Outcomes	
Recurrence	25 (29.1%)
Death	17 (20.0%)

^a^T, tumor size; N, lymph node status; SBR, Scarff-Bloom-Richardson histological grade; ER, estrogen receptor; PR, progesterone receptor.

### DNA extraction and gene quantification

Each frozen tumor specimen was pulverized in liquid nitrogen with an automatic grinder (Cryobroyeur 2000P Automatique; Rivoire, Montpellier, France), homogenized in a Polytron homogenizer in buffer (20 mM Tris HCI, 1.5 mM ethylenediaminetetraacetic acid (EDTA), 10 mM Na_2_MoO_4_, 1.5 mM dithiothreitol and 10% glycerol, pH 7.4) (buffer:tissue ratio was 10:1 (vol/wt)) used for ER and PR measurement and centrifuged at 10,000 × *g *for 15 minutes. Total genomic DNA was extracted from the pellet. The DNA concentration was determined by measuring the absorbance at 260 nm, and all samples had a 260/280-nm ratio higher than 1.7. DNA was stored at -20°C in TE buffer (10 mM Tris and 0.5 mM EDTA, pH 7.6). All procedures were carried out at 4°C unless noted otherwise. qPCR amplifications were performed with 10 ng of DNA on a Rotorgene 6000 apparatus (Qiagen, Courtaboeuf, France) using ABsolute Blue qPCR SYBR Green Mix (ref AB-4167; ThermoFisher Scientific, Illkirch, France) with an initial 15-minute denaturation step at 95°C followed by 40 cycles. The cycling temperatures and the primers used for each gene are summarized in Table 2. After completion of the amplification, samples were subjected to a temperature ramp (from the annealing temperature to 95°C with a transition rate of 0.1°C/s) with continuous fluorescence monitoring for melting curve analysis. *MED1, STARD3, HER2, GRB7, THRA, RARA, TOP2A, IGFBP4, CCR7, KRT20*, *KRT19 *and *GAS *amplification levels were normalized to those of the somatostatin receptor type II (*SSTR2*) gene that is localized on Chr17q24. *HER2 *quantification was also normalized to the expression values of glyceraldehyde 3-phosphate dehydrogenase (*GAPDH*), which is localized on chromosome 12p13, and of β-actin (*ACBT*), which is localized on chromosome 7p22, as controls for polysomy. *SSTR2 *quantification was also normalized to *GAPDH *and *ACBT *to detect *SSTR2 *amplification. Quantification was obtained by constructing a standard curve from serial dilutions of normal genomic DNA (ref 11 691 112 001; Roche, Meylan, France). For *HER2 *amplification, genomic DNA from SKBR3 cells was used as a positive control and DNA from MCF-7 cells was used as a negative control. Water was used as a negative control for PCR contamination. A gene was considered to be amplified when the target gene-to-reference gene ratio was ≥2, and it was considered to be deleted when the target gene-to-reference gene ratio was ≤0.5.

**Table 2:** Primer sequences and qPCR conditions used to quantify *MED1, STARD3, HER2, GRB7, THRA, RARA, TOP2A, IGFBP4, CCR7, KRT20, KRT19, GAS, ACBT, GAPDH *and *SSTR2*^a^

GenBank accession number	Gene name/primer	Sequence 5'-3'	Size nt	GC, %	**Amplicon**, bp	Annealing temperature
NP_004765	*MED1 *sense	ATT CTC CTG GGC TTC TCC AA	20	50%	97	54°C
	*MED1 *antisense	CCA CAC ACC AGG GAG TCA TT	20	55%		
NP_006795	*STARD3 *sense	CAG GCT GCT AGG GTG TAA CTG	21	57%	114	64°C
	*STARD3 *antisense	GAC AGA GCA CCG GAG AAC AG	20	60%		
NM_004448	*HER2 *sense	GCT CCC CAT ATG TCT CCC G	19	63%	101	58°C
	*HER2 *antisense	CCG GAC ATG GTC TAA GAG GC	20	60%		
NP_005301	*GRB7 *sense	CTC TGG CTC AGA ACT TCC TGA AT	23	48%	142	60°C
	*GRB7 *antisense	GTG CCC TTG GTG GAG TAA TAG AG	23	52%		
NP_003241	*THRA *sense	GTG GAC AAG ATC GAG AAG AGT CAG	24	50%	119	60°C
	*THRA *antisense	AGG TCA GTC ACC TTC ATC AGC AG	23	52%		
NP_000955	*RARA *sense	GGA GTG CTC AGA GTG GGT TC	20	60%	110	64°C
	*RARA *antisense	AGA AGG TCA TGG TGT CCT GCT C	22	55%		
NP_001058	*TOP2A *sense	GAT TCT GGA CCA ACC TTC AAC TA	23	43%	121	58°C
	*TOP2A *antisense	ATG TAC CAT CCT ACT ATC AAC TCA CTT T	28	36%		
NP_001543	*IGFBP4 *sense	CTC TTC CGG TGC TGA CCT CT	20	60%	146	60°C
	*IGFBP4 *antisense	GGT GCT CCG GTC TCG AAT	18	61%		
NP_001829	*CCR7 *sense	AGG CTA AAT CCC AGC CAG AG	20	55%	127	58°C
	*CCR7 *antisense	CTG TGG TGT TGT CTC CGA TG	20	55%		
XP_352920	*KRT20 *sense	ATG GCT TCA GAA GGA CCA GTT	21	48%	95	56°C
	*KRT20 *antisense	TGG AGA TCA GCT TCC ACT GTT A	22	45%		
NP_002267	*KRT19 *sense	TGA CAT GCG AAG CCA ATA TG	20	45%	124	56°C
	*KRT19 *antisense	AAA GCC CTC CCC TTC CTA AC	20	55%		
X00183	*GAS *sense	TCT CCC CAG ACT GGC TCT GA	20	60%	146	64°C
	*GAS *antisense	GCC GAA GTC CAT CCA TCC AT	20	55%		
NP_001041	*SSTR2 *sense	GCC TCC AGG GTC CAT TAA GG	20	60%	101	60°C
	*SSTR2 *antisense	ATT GAG TGG CTC ATC CGC C	19	58%		
NP_001092	*ACBT *sense	CCA CAC TGT GCC CAT CTA CG	20	60%	99	65°C
	*ACBT *antisense	AGG ATC TTC ATG AGG TAG TCA GTC AG	26	46%		
NT_009759	*GAPDH *sense	CTC ACG TAT TCC CCC AGG TT	20	55%	161	58°C
	*GAPDH *antisense	CCC AAA GCA CAT TTC TTC CA	20	45%		

^a^qPCR, quantitative polymerase chain reaction; *MED1*, mediator complex subunit 1 gene; *STARD3*, steroidogenic acute regulatory-related lipid transfer (START) domain containing 3 gene; *HER2*, human epidermal growth factor receptor 2 gene; *GRB7*, growth factor receptor-bound protein 7 gene; *THRA*, thyroid hormone receptor α gene; *RARA*, retinoic acid receptor α gene; *TOP2A*, topoisomerase IIα gene; *IGFBP4*, insulin-like growth factor-binding protein 4 gene; *CCR7*, C-C chemokine receptor type 7 gene; *KRT20*, cytokeratin 20 gene; *KRT19*, cytokeratin 19 gene; *GAS*, gastrin gene; *ACBT*, β-actin gene; *GAPDH*, glyceraldehyde 3-phosphate dehydrogenase gene; *SSTR2*, somatostatin receptor type II gene.

### Statistical analysis

Categorical variables were described using frequency distributions, and continuous variables were stated using medians and ranges. Correlations between genes was evaluated using the Spearman's ρ correlation. Association between categorical variables was assessed with the χ^2 ^test (or Fisher's exact test when appropriate). Differences were considered statistically significant when *P *< 0.05. Survival rates were estimated from the date of surgery to the date of the event of interest using the Kaplan-Meier method. The median survival was calculated with 95% confidence interval (95% CI). For overall survival (OS), the event of interest was death, regardless of the cause. Patients lost to follow-up were censored at the last documented visit. For relapse-free survival (RFS), the event of interest was recurrence. Patients who were alive at the last follow-up examination and without recurrence were censored at the time of the last follow-up examination. Patients who died without recurrence were censored at the date of death. Differences in survival rates were compared using a log-rank test. Statistical analyses were performed with Stata 10.0 software (Stata Corp., College Station, TX, USA).

## Results

### Description of the study population

Eighty-six patients with HER2-amplified breast cancer were included in the study. The median follow-up period was 55 months (range, 6 to 81 months). A total of 25 patients (29%) relapsed after a median of 18 months from the date of surgery (range, 2 to 71 months), and 17 patients (20%) died after a median of 31 months (range, 6 to 78 months). The 5-year RFS rate was 69% (95% CI, 57% to 78%) and the 5-year OS rate was 79% (95% CI, 67% to 87%). There were no statistical associations between RFS or OS and the adjuvant treatment administered. The distribution of tumors relative to their pattern of amplified and nonamplified genes was homogeneous in the different treatment groups.

### Analysis of the Chr17q12-q21 amplicon by qPCR

**Figure 1:**
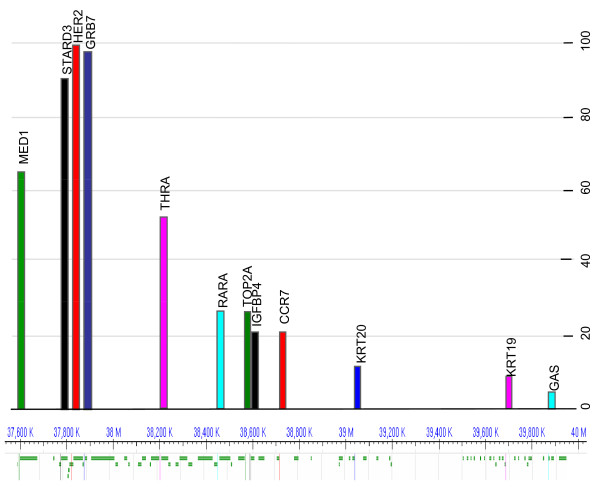
**Gene amplification distribution on chromosome 17q12-q21 in Human epidermal growth factor receptor 2 (HER2)-positive breast cancer specimens on the basis of the overview of chromosome 17 with the National Center for Biotechnology Information's Sequence Viewer version 2.9 database **[55]. The frequency of amplification (percentage of positive breast cancer specimens on the *y*-axis) for a gene decreases with the increase of its distance from HER2. *MED1*, mediator complex subunit 1 gene; *STARD3*, steroidogenic acute regulatory-related lipid transfer (START) domain containing 3 gene; *HER2*, human epidermal growth factor receptor 2 gene; *GRB7*, growth factor receptor-bound protein 7 gene; *THRA*, thyroid hormone receptor α gene; *RARA*, retinoic acid receptor α gene; *TOP2A*, topoisomerase IIα gene; *IGFBP4*, insulin-like growth factor-binding protein 4 gene; *CCR7*, C-C chemokine receptor type 7 gene; *KRT20*, cytokeratin 20 gene; *KRT19*, cytokeratin 19 gene; *GAS*, gastrin gene.

The variability in the efficiency of each parameter was between 92% and 105% with a standard deviation of 0.04. The reference genes (*SSTR2*, *GAPDH *and *ACBT*) were chosen in regions previously described as presenting a low level of instability or amplification [25,26] and were controlled together to detect amplification. Their crossing point values were 19.2 ± 0.56, 18.7 ± 0.47 and 17.6 ± 0.43, respectively. The absence of *SSTR2 *amplification was confirmed in all samples by measuring the *SSTR2*:*GAPDH *and *SSTR2*:*ACBT *ratios, which in all cases were <1.5. Finally, none of the 40 HER2-negative cancer specimens (negative controls) showed amplification of the studied genes. The status (amplified, deleted or normal) of the 11 genes (*MED1, STARD3, GRB7, THRA, RARA, TOP2A, IGFBP4, CCR7, KRT20*, *KRT19 *and *GAS*) evaluated in the 86 HER2-positive cancer samples is summarized in Table 3. Coamplification with *HER2 *occurred in 65.1% of samples for *MED1 *(56 of 86), 93% for *STARD3 *(80 of 86), 97.7% for *GRB7 *(84 of 86), 54.7% for *THRA *(47 of 86), 26.7% for *RARA *(23 of 86), 26.7% for *TOP2A *(23 of 86), 20.9% for *IGFBP4 *(18 of 86), 20.9% for *CCR7 *(18 of 86), 14% for *KRT20 *(12 of 86), 11.6% for *KRT19 *(10 of 86) and 7% for *GAS *(6 of 86). *HER2 *presented the highest level of amplification (mean, 6.4; range, 2 to 32), whereas the frequency of amplification of the other genes decreased according to the distance of the gene from *HER2 *(Figure 1). The amplification levels of *HER2 *and of the other genes were compared by linear regression analysis. *HER2 *amplification levels were correlated only with the amplification levels of *MED1 *(ρ = 0.84), *STARD3 *(ρ = 0.87) and *GRB7 *(ρ = 0.93), but not of other genes, particularly *TOP2A *(ρ = 0.65). Gene deletions occurred in fewer than 5.8% of the samples, regardless of the gene studied (Table 3).

**Table 3:** Gene amplification quantification relative to the *SSTR2 *reference gene^a^

Gene	Number of patients (%) (*n *= 86)	Median (range)
*MED1*		
Deletion	3 (3.5%)	0.5 (0.5 to 0.5)
Normal	27 (31.4%)	0.9 (0.6 to 1.9)
Amplification	56 (65.1%)	4.6 (2 to 27.8)
Total	86 (100.0%)	2.6 (0.5 to 27.8)
*STARD3*		
Deletion	0	-
Normal	6 (7.0%)	1.5 (0.7 to 1.9)
Amplification	80 (93.0%)	6.4 (2.0 to 74.9)
Total	86 (100.0%)	5.9 (0.7 to 74.9)
*HER2*		
Amplification	86 (100.0%)	6.4 (2 to 32)
*GRB7*		
Deletion	0	-
Normal	2 (2.3%)	1.1 (0.6 to 1.7)
Amplification	84 (97.7%)	6.8 (2.3 to 36.5)
Total	86 (100.0%)	6.6 (0.6 to 36.5)
*THRA*		
Deletion	1 (1.2%)	0.5
Normal	38 (44.2%)	1.1 (0.6 to 1.9)
Amplification	47 (54.7%)	4.4 (2.0 to 11.7)
Total	86 (100.0%)	2.2 (0.5 to 11.7)
*RARA*		
Deletion	3 (3.5%)	0.5 (0.4 to 0.5)
Normal	60 (69.8%)	1.0 (0.6 to 1.9)
Amplification	23 (26.7%)	3.4 (2.0 to 11.1)
Total	86 (100.0%)	1.1 (0.4 to 11.1)
*TOP2A*		
Deletion	4 (4.7%)	0.5 (0.5 to 0.5)
Normal	59 (68.6%)	1.0 (0.6 to 1.8)
Amplification	23 (26.7%)	3.2 (2 to 7)
Total	86 (100.0%)	1.1 (0.5 to 7)
*IGFBP4*		
Deletion	5 (5.8%)	0.5 (0.5 to 0.5)
Normal	63 (73.3%)	1.0 (0.6 to 1.9)
Amplification	18 (20.9%)	3.1 (2 to 8)
Total	86 (100.0%)	1.1 (0.5 to 8)
*CCR7*		
Deletion	3 (3.5%)	0.4 (0.4 to 0.5)
Normal	65 (75.6%)	0.9 (0.6 to 1.8)
Amplification	18 (20.9%)	3.3 (2 to 14.9)
Total	86 (100.0%)	1.0 (0.4 to 14.9)
*KRT20*		
Deletion	2 (2.3%)	0.5 (0.5 to 0.5)
Normal	72 (83.7%)	1.1 (0.6 to 1.8)
Amplification	12 (14.0%)	3.1 (2.3 to 11.6)
Total	86 (100.0%)	1.1 (0.5 to 11.6)
*KRT19*		
Deletion	5 (5.8%)	0.5 (0.5 to 0.5)
Normal	71 (82.6%)	1.0 (0.6 to 1.8)
Amplification	10 (11.6%)	3.0 (2 to 6.4)
Total	86 (100.0%)	1.0 (0.5 to 6.4)
*GAS*		
Deletion	2 (2.3%)	0.5 (0.4 to 0.5)
Normal	78 (90.7%)	1.0 (0.6 to 1.7)
Amplification	6 (7.0%)	2.8 (2 to 14.4)
Total	86 (100.0%)	1.0 (0.4 to 14.4)

^a^Amplification: target gene/*SSTR2 *ratio ≥2; deletion: target gene/*SSTR2 *ratio ≤0.5; normal: 0.5 < target gene/*SSTR2 *ratio <2. *MED1*, mediator complex subunit 1 gene; *STARD3*, steroidogenic acute regulatory-related lipid transfer (START) domain containing 3 gene; *HER2*, human epidermal growth factor receptor 2 gene; *GRB7*, growth factor receptor-bound protein 7 gene; *THRA*, thyroid hormone receptor α gene; *RARA*, retinoic acid receptor α gene; *TOP2A*, topoisomerase IIα gene; *IGFBP4*, insulin-like growth factor-binding protein 4 gene; *CCR7*, C-C chemokine receptor type 7 gene; *KRT20*, cytokeratin 20 gene; *KRT19*, cytokeratin 19 gene; *GAS*, gastrin gene; *SSTR2*, somatostatin receptor type II gene.

### Relation between the pattern of gene amplification and clinical characteristics

**Figure 2:**
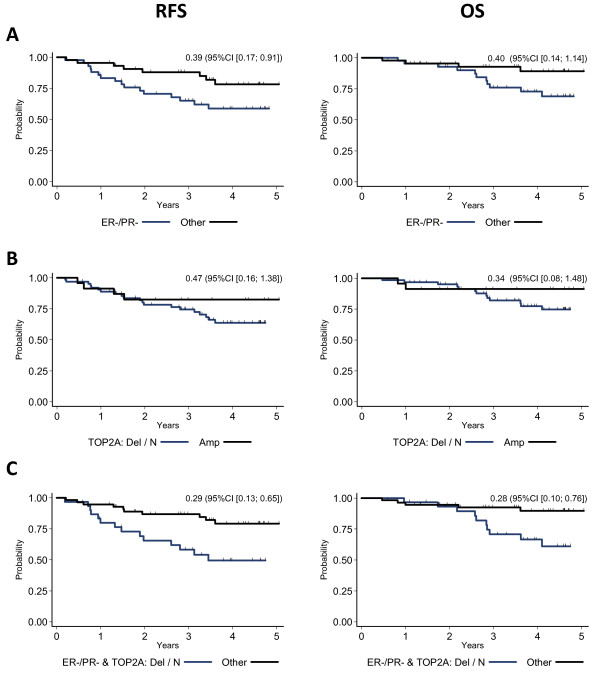
**Kaplan-Meier curves for relapse-free survival (RFS) and overall survival (OS)**. **(a) **Estrogen receptor/progesterone receptor (ER/PR) status. Blue line indicates ER- and PR-negative (ER^-^/PR^-^) status; black line indicates ER-, PR- or ER/PR-positive (ER^-^/PR^+^, ER^+^/PR^- ^or ER^+^/PR^+^) status. **(b) **Topoisomerase IIα (*TOP2A*) amplification. Blue line indicates deleted or nonamplified (normal) *TOP2A *(TOP2A Del/N); black line indicates amplified *TOP2A *(TOP2A Amp). For RFS, *P *= 0.159; for OS, *P *= 0.132. **(c) ***TOP2A *and ER/PR status. Blue line indicates ER- and PR-negative (ER^-^/PR^-^) and deleted or nonamplified *TOP2A *(TOP2A Del/N); black line indicates ER-, PR-, or ER/PR-positive (ER^-^/PR^+^, ER^+^/PR^- ^and ER^+^/PR^+^) status and amplified *TOP2A *(TOP2A Amp). For RFS, *P *= 0.001; for OS, *P *= 0.008.

Node status (N), Scarff-Bloom-Richardson histological (SBR) grade and ER/PR-negative status were significantly associated with worse outcomes in terms of both RFS rate (*P *= 0.007, *P *= 0.004 and *P *= 0.024, respectively) and OS rate (*P *= 0.020, *P *= 0.048 and *P *= 0.076, respectively) (univariate analysis) (see Table 4 and Figure 2 for ER/PR status). The patterns of gene amplification were not significantly correlated with the T or SBR grade. Conversely, *MED1 *amplification tended to be associated with ER-positive tumors (*P *= 0.072), whereas amplification of *RARA*, *KRT20 *or *KRT19 *was significantly associated with node-positive breast cancers (*P *= 0.030, *P *= 0.002 and *P *= 0.033, respectively). Moreover, patients with TOP2A-amplified tumors tended to have better RFS rates (hazard ratio (HR) = 0.47, 95% CI 0.16 to 1.38, *P *= 0.159) and OS rates (HR = 0.34, 95% CI 0.08 to 1.48, *P *= 0.132) than patients with TOP2A nonamplified tumors (Figure 2). The subgroup of patients with ER/PR-negative cancers without amplification of *TOP2A *in particular had the worst RFS rates (HR = 0.29, 95% CI 0.13 to 0.65, *P *= 0.001) and the worst OS rates (HR = 0.28, 95% CI 0.10 to 0.76, *P *= 0.008).

**Table 4:** Univariate analysis of the different variables^a^

		RFS			OS		
		
Variable	Number of patients	Events	5-year RFS	HR (95% CI)	Events	5-year OS	HR (95% CI)
T							
1	35	8	74.2	1	6	84.2	1
2	46	15	65.9	1.51 (0.64 to 3.58)	10	74.3	1.30 (0.47 to 3.60)
3 or 4	5	2	53.3	2.15 (0.45 to 10.4)	1	80.0	1.34 (0.16 to 11.3)
		*P *= 0.510*			*P *= 0.868*		
N							
N^-^	35	4	88.1	1	2	94.2	1
N^+^	51	21	55.8	3.94 (1.35 to 11.5)	15	70.0	4.89 (1.12 to 21.4)
		*P *= 0.007*			*P *= 0.020*		
SBR							
2	36	5	84.9	1	4	89.4	1
3	49	20	55.8	3.80 (1.42 to 10.2)	13	70.8	2.96 (0.96 to 9.15)
		*P *= 0.004*			*P *= 0.048*		
ER							
ER^-^	60	19	66.9	1	13	76.2	1
ER^+^	26	6	70.1	0.64 (0.25 to 1.60)	4	84.9	0.68 (0.22 to 2.10)
		*P *= 0.333*			*P *= 0.505*		
PR							
PR^-^	44	18	57.6	1	13	67.3	1
PR^+^	42	7	80.9	0.36 (0.15 to 0.87)	4	92.4	0.32 (0.10 to 0.99)
		*P *= 0.018*			*P *= 0.037*		
ER/PR							
ER^-^/PR^-^	42	17	58.7	1	12	68.9	1
Other	44	8	78.2	0.39 (0.17 to 0.91)	5	89.2	0.40 (0.14 to 1.14)
		*P *= 0.024*			*P *= 0.076*		
*MED1*							
Del/Nm	30	10	66.2	1	7	72.9	1
Amp	56	15	70.0	0.76 (0.34 to 1.70)	10	82.5	0.63 (0.23 to 1.69)
		*P *= 0.507*			*P *= 0.354*		
*STARD3*							
Del/Nm	6	2	55.6	1	2	53.3	1
Amp	80	23	69.4	0.85 (0.20 to 3.62)	15	80.9	0.48 (0.11 to 2.14)
		*P *= 0.826*			*P *= 0.327*		
*GRB7*							
Del/Nm	2	1	50.0	1	1	0.00	1
Amp	84	24	69.3	0.30 (0.04 to 2.33)	16	80.1	0.15 (0.02 to 1.20)
		*P *= 0.225*			*P *= 0.038*		
*THRA*							
Del/Nm	39	16	59.0	1	11	73.8	1
Amp	47	9	79.3	0.50 (0.22 to 1.13)	6	84.7	0.53 (0.19 to 1.43)
		*P *= 0.090*			*P *= 0.199*		
*RARA*							
Del/Nm	63	21	64.1	1	15	75.2	1
Amp	23	4	82.4	0.50 (0.17 to 1.45)	2	91.3	0.36 (0.08 to 1.59)
		*P *= 0.190*			*P *= 0.162*		
*TOP2A*							
Del/Nm	63	21	63.7	1	15	74.8	1
Amp	23	4	82.4	0.47 (0.16 to 1.38)	2	91.3	0.34 (0.08 to 1.48)
		*P *= 0.159*			*P *= 0.132*		
*IGFBP4*							
Del/Nm	68	21	66.6	1	15	76.9	1
Amp	18	4	77.4	0.70 (0.24 to 2.06)	2	88.9	0.49 (0.11 to 2.16)
		*P *= 0.520*			*P *= 0.335*		
*CCR7*							
Del/Nm	68	21	67.0	1	16	75.3	1
Amp	18	4	75.4	0.66 (0.22 to 1.93)	1	94.4	0.21 (0.03 to 1.61)
		*P *= 0.442*			*P *= 0.099*		
*KRT20*							
Del/Nm	74	21	69.2	1	15	78.8	1
Amp	12	4	66.7	1.31 (0.45 to 3.81)	2	81.5	1.02 (0.23 to 4.49)
		*P *= 0.622*			*P *= 0.981*		
*KRT19*							
Del/Nm	76	23	67.6	1	16	78.3	1
Amp	10	2	78.7	0.70 (0.16 to 2.96)	1	90.0	0.64 (0.08 to 4.82)
		*P *= 0.624*			*P *= 0.658*		
*GAS*							
Del/Nm	80	24	68.0	1	16	79.1	1
Amp	6	1	83.3	0.64 (0.09 to 4.74)	1	83.3	1.21 (0.16 to 9.20)
		*P *= 0.660*			*P *= 0.852*		
							
ER^-^/PR^- ^and *TOP2A *Del/Nm	30	15	49.2	1	11	61.0	1
Others	56	10	79.2	0.29 (0.13 to 0.65)	6	89.7	0.28 (0.10 to 0.76)
		*P *= 0.001*			*P *= 0.008*		

^a^T, tumor size; N, lymph node status; SBR grade, Scarff-Bloom-Richardson histological grade; ER, estrogen receptor; PR, progesterone receptor; Del, deletion; Nm, normal; Amp, amplification; RFS, relapse-free survival; OS, overall survival; HR, hazard ratio; 95% CI, 95% confidence interval; *MED1*, mediator complex subunit 1 gene; *STARD3*, steroidogenic acute regulatory-related lipid transfer (START) domain containing 3 gene; *HER2*, human epidermal growth factor receptor 2 gene; *GRB7*, growth factor receptor-bound protein 7 gene; *THRA*, thyroid hormone receptor α gene; *RARA*, retinoic acid receptor α gene; *TOP2A*, topoisomerase IIα gene; *IGFBP4*, insulin-like growth factor-binding protein 4 gene; *CCR7*, C-C chemokine receptor type 7 gene; *KRT20*, cytokeratin 20 gene; *KRT19*, cytokeratin 19 gene; *GAS*, gastrin gene; *SSTR2*, somatostatin receptor type II gene; *log-rank test.

## Discussion

In this work, we quantified gene amplification in the 17q12 to 17q21 region around *HER2 *in HER2-amplified breast tumors. Among the patients with primary breast cancer, fewer than 12% had HER2-positive tumors. This result is in accordance with recent studies reporting 12% to 15% of *HER2 *amplification compared to the 20% to 25% found in historical studies [29,30], in which Chr17 polysomy may have led to discordant interpretations between higher signals due to polysomy and those due to an absolute increase of *HER2 *gene copy number in a significant proportion of cases [31]. Gene amplification on the centromeric side (*MED1 *and *STARD3*) and the distal side (*GRB7*, *THRA*, *RARA*, *TOP2A*, *IGFBP4*, *CCR7*, *KRT20*, *KRT19 *and *GAS*) relative to *HER2 *localization on Chr17q was determined by qPCR, an efficient method for performing copy number analysis [32], and with reference genes located on Chr17 (*SSTR2*) and Chr4 and Chr2 (*GAPDH *and *ACBT*, respectively) to differentiate polysomy from true amplification. In fact, tumors presenting only Chr17 polysomy should not be considered as HER2-amplified, and patients with this presentation might not benefit from anti-HER2 therapy, although these tumors present moderate HER2 overexpression [28,33], but only as a result of treatment with anthracyclines [34].

We have confirmed that gene amplification, including that of *TOP2A*, occurs only in patients with HER2-amplified breast tumors. We then showed that *HER2 *presents the highest amplification value and that the frequency of amplification of a gene decreases with its distance from *HER2*. These results suggest that instability within Chr17q is driven by *HER2 *amplification. Indeed, HER2-positive tumors have higher levels of overall genomic instability than HER2-negative tumors, supporting the idea that *HER2 *amplification is the key amplicon driver on Chr17q [35,36]. Moreover, we found a correlation between the amplification levels of *HER2 *and that of *MED1*, STARD3 and GRB7, but not of *TOP2A *and the other genes localized on the distal side of Chr17q. Therefore, the SRA of *HER2 *seems to be limited to a small number of genes, among which are *STARD3 *and *GRB7 *as described by Katoh *et al. *[37] and as recently reported by Beroukhim *et al. *[38] in different cancer types. Kuwahara *et al. *[39] identified a region between *HER2 *and *GRB7 *(a gene located 47 kb from *HER2*) that contains a recombinant hot spot for amplification, deletion and translocation. This site might be located between *GRB7 *and *THRA*, a gene located 315 kb from *GRB7*, given that 84 (97.7%) of the cancer specimens we assessed presented GRB7 amplification, whereas only 47 (54.7%) showed *THRA *amplification. The presence of this hot spot might explain why the frequency of recombination for a gene is higher when it is located closer to *HER2 *and gradually decreases for genes located far away from *HER2*. In general, the gene amplification architectures derived from our results illustrate the complexity of the rearrangement mechanisms (sister chromatid breakage-fusion-bridge cycles, formation and reinsertion of double minutes and repeated units at a single locus) and highlight the diversity of HER2-amplified cancers [40,41].

Finally, we found a low overall number of deletions (<5.8%), regardless of the gene analyzed. For *TOP2A*, the deletion rate was 4.7%, which is low compared with values found in other studies (between 8.1% and 35%) [22]. This might be explained by the fact that in heterogenic tumors, which simultaneously present amplifications and deletions, qPCR cannot detect deletions that are present in a very small number of cells. It is also possible that in certain studies the percentage of tumors with deletions was overestimated as a result of poor standardization of the FISH technique. Indeed, Di Leo *et al. *[42,43] recently showed high interlaboratory variations for *TOP2A *quantification, whereas Press *et al. *[44] found a deletion rate comparable to ours in a prospective study using standardized FISH.

We then investigated the clinical impact of Chr17 anomalies. Amplification of *RARA*, *KRT20 *and *KRT19 *correlated with node invasion, suggesting that tumors with amplification of genes on the distal side of Chr17q are more aggressive than tumors with amplifications restricted to the SRA of *HER2*. *MED1 *amplification showed a tendency to be associated with ER-positive tumors. *MED1 *encodes the mediator complex subunit 1 (MED1) [45] that anchors mediator to ERβ [46] while also interacting with ERα [47]. Although our study did not show any significant impact of *MED1 *amplification on survival, the prognostic value of *MED1 *should nevertheless be evaluated in a larger ER/PR-positive population. Similarly, *TOP2A *amplification was not a statistically significant prognostic factor, but patients with tumors in which *TOP2A *was amplified tended to have better RFS and OS rates (see Figure 2b). This result is in accord with a recent study in which patients with HER2-positive, TOP2A-amplified cancers presented a trend of better survival than patients with HER2-positive breast cancers with deleted or normal *TOP2A *[48]. Conversely, the subgroup of patients with ER/PR-negative breast cancers and nonamplified (normal or deleted) *TOP2A *showed the least favorable RFS and OS rates in our population. We could not determine whether the prognosis was even less favorable in the case of ER/PR-negative tumors with TOP2A-deleted status than of tumors with normal *TOP2A*. Nevertheless, *TOP2A *deletions, which are often heterozygous [49], do not result in total loss of expression of the protein [50]. Their unfavorable prognosis might be explained by the fact that ER/PR-negative, TOP2A nonamplified tumors may not be responsive to hormone therapy and/or to anthracycline-based therapy because of the absence or insufficient number of targets. Adjuvant therapy would thus not be beneficial for these patients. Indeed, recent findings suggest that the presence of *TOP2A *deletions is not associated with better chemosensitivity or response to anthracyclines, while the occurrence of *TOP2A *amplifications may identify a subgroup of patients with increased response to anthracyclines [18,44]. Many studies have been conducted on the impact of the presence of *HER2 *and *TOP2A *amplifications on survival and response to chemotherapy. Only a few studies found that there was no benefit to treating patients with HER2-positive cancer with anthracyclines or that *TOP2A *amplification had no predictive value. The subject remains controversial [51], but nevertheless two large trials (the National Surgical Adjuvant Breast and Bowel Project B-11 and the National Cancer Institute of Canada MA.5 studies) have retrospectively shown a correlation between *HER2 *amplification and benefit from adjuvant anthracycline-based chemotherapy [52,53]. Moreover, a pooled analysis that included 8 studies and 1,536 HER2-positive patients indicated that anthracyclines were superior to non-anthracycline-based regimens in terms of disease-free and overall survival [54].

## Conclusions

Few studies have used qPCR to assess gene copy number alterations in HER2-amplified breast cancer. The assay we have developed can be used to assess the amplification status of multiple genes. We have shown that *MED1*, *STARD3*, *TOP2A*, *GRB7*, *THRA*, *RARA*, *IGFBP4*, *CCR7*, *KRT19*, *KRT20 *and *GAS *amplification occurred only in HER2-amplified breast cancers with variable frequencies. We have confirmed that *HER2 *seems to drive genomic instability along Chr17q and that *TOP2A *was not included in the SRA of *HER2*. The different patterns of amplification we observed suggest complex amplification mechanisms leading to a variety of subtypes. *RARA*, *KRT19 *and *KRT20 *amplification could be related to a more invasive breast cancer profile. Moreover, we confirm the clinical importance of identifying the subgroup of patients with ER/PR-negative, TOP2A nonamplified cancer, as they have the worst prognosis. A prospective study including a larger number of patients treated with trastuzumab and anthracycline-based adjuvant therapy would be useful to determine the predictive value of *TOP2A *amplification and the prognostic value of gene copy number variations along Chr17q.

## Abbreviations

*ACBT*: β-actin; *CCR7*: C-C chemokine receptor type 7; Chr17q: chromosome 17q; ER: estrogen receptor; FISH: fluorescence *in situ *hybridization; *GAPDH: glyceraldehyde 3-phosphate dehydrogenase; GAS: gastrin; GRB7*: growth factor receptor-bound protein 7; *HER2*: human epidermal growth factor receptor 2; *IGFBP4*: insulin-like growth factor-binding protein 4; *KRT19*: cytokeratin 19; *KRT20*: cytokeratin 20; *MED1*: mediator complex subunit 1; N: nodal status; OS: overall survival; PR: progesterone receptor; qPCR: quantitative polymerase chain reaction; *RARA*: retinoic acid receptor α; RFS: relapse-free survival; SBR grade: Scarff-Bloom-Richardson histological grade; SRA: small region of amplification; *SSTR2*: somatostatin receptor type II; *STARD3*: steroidogenic acute regulatory-related lipid transfer (START) domain containing 3; *THRA*: thyroid hormone receptor α; *TOP2A*: topoisomerase IIα; T: tumor size.

## Competing interests

The authors declare that they have no competing interests.

## Authors' contributions

PJL contributed to the conception and design of the entire study, coordinated sample collection, interpreted data and drafted the manuscript. FF made the first design of the quantitative polymerase chain reaction (qPCR) assays, contributed to data interpretation as well as statistical analysis and assisted in drafting the manuscript. CMB supervised the statistical analysis and assisted in drafting the manuscript. ACL was responsible for designing and optimizing the qPCR assays and carried out most of the experiments with the DNA samples described in this paper. PMM and LO made original observations leading to this work and contributed to the critical revision of the manuscript. WJ selected the eligible patients, acquired the clinicopathological data and contributed to the drafting of the manuscript. All authors read and approved the final manuscript.
